# Solar panels bring power to rural health facilities

**DOI:** 10.2471/BLT.23.021123

**Published:** 2023-11-01

## Abstract

Increasingly affordable solar power has the potential to transform health service delivery if investors can get behind it. Fid Thomson reports.

Dr Bwire Chirangi was operating on a woman suffering from a ruptured ectopic pregnancy when the lights went out.

“Suddenly everything went dark,” recalls the Medical Director of the Shirati KMT Hospital in Shirati, Mara region, United Republic of Tanzania.

“I told the anaesthesiologist to start the back-up generator, but it didn’t work,” he says. “I had to finish the operation by the light of my assistant’s cell phone.”

While surgery may provide the most hair-raising examples of how electricity outages can affect health care, a range of services – from the basic to the sophisticated – are impacted.

Dr Lia Tadesse, Minister of Health of Ethiopia, spells it out: “Without power, health facilities cannot operate life-saving equipment such as incubators, refrigerate the vaccines and medical products that require cold storage, or run basic diagnostic lab equipment such as imaging machines and blood analysers. Basically, we need it for everything.”

Inadequate power supply is a problem for health workers and patients in low- and middle- income countries worldwide. Indeed, an estimated 1 billion people in such countries are obliged to use health care in facilities with unreliable power, or no power at all. But countries in the African Region are particularly affected.

According to *Energizing health: accelerating electricity access in health-care facilities,* released in January 2023 by the World Health Organization (WHO) and partners, some 41% (68 350/166 720) of health-care facilities situated across the 41 low- and lower-middle income countries of the African Region lack reliable access to electricity grids, while at least 15% (25 000) have no access whatsoever.

Until recently, the main obstacle to reliable power was the cost of installing, extending and maintaining electrical grids. Now, because of increasingly affordable solar power, that picture is changing.

“The average cost of crystalline photovoltaic modules has decreased by 90% in the last ten years, while the cost of batteries, essential for storing the electricity generated during the day, has decreased as much as 70%,” says Salvatore Vinci, a technical officer and expert on sustainable energy at WHO.

According to Vinci, price comparisons with fossil fuel alternatives are challenging due to the variables in different countries but, as an example, he cites WHO's recent work in Somalia on solarization of health-care facilities.

“The cost of a 5 kW solar capacity system coupled with battery in Somalia is around 33 000 United States dollars (US$). If we add the cost of battery replacement and of operation and maintenance for 20 years, this cost can go up to US$ 48 000. In comparison, the cost of electricity based on diesel generators in the country, covering the same energy requirements over the same period, would be about US$ 116 000.”

“[It’s] crucial to get away from the…. install-and-forget approach.”

Dalila Gonçalves

For Vinci, the case for solarization is clear. “At current prices, areas with limited or no grid electricity now have the option of leapfrogging directly to a decentralized, sustainable and clean energy source,” he says.

To date, initiatives have tended to focus on boosting capacity in specific areas, such as immunization. In Ethiopia, for example, where only around one third of the 17 000 community health posts (often simple two-room buildings in rural areas) are connected to electricity, solar power refrigerators have been installed in 10 000.

“Our health posts are key to providing immunization services, so refrigeration was a priority,” explains Tadesse, adding that the health ministry has also installed complete solar systems in 1000 of the health posts.

Neighbouring Somalia has also started to use solar energy in the health sector and, in August 2023, opened its first national blood bank powered by solar panels, with support from WHO and United Nations partners.

Dr Mohamed Jama, senior policy advisor to Somalia’s Minister of Health, is hopeful that further initiatives will follow. “I foresee a major change in the expansion of immunization services, supported by reliable refrigeration,” he says, noting that up to 30% of infant and child deaths in Somalia are vaccine-preventable.

While such applications clearly make a difference, Vinci is keen to see more whole-of-facility initiatives, such as those launched in a multi-country project backed by WHO, Gavi, the Vaccine Alliance, and the United Nations Children’s Fund (UNICEF) in 2023.

“The project involves the complete solar electrification of 1 000 health-care facilities across Ethiopia, Pakistan, Uganda and Zambia,” Vinci says, adding that WHO and UNICEF, with the technical support of the SELCO Foundation, plan to further expand solarization efforts in the coming years, and to electrify 10 000 primary health centres across resource-constrained regions, while at the same time providing them with essential medical equipment.

The SELCO Foundation has already established itself as a key stakeholder in solar power, having worked on a project to solarize some 2 000 public health facilities in India between 2021–2022, and is currently supporting the health ministry and partners in bringing solar energy to 25 000 health facilities in 12 Indian states by 2026.

In many instances, the solarization of health facilities is happening as part of broader initiatives to establish so-called mini grids. This is true, for example, of the rural renewable energy project that is being implemented in Sierra Leone on behalf of the government by the United Nations Office for Project Services (UNOPS).

According to Dalila Gonçalves, acting Regional Director for Africa at UNOPS, the project has so far brought solar power to 95 communities, powering households, schools and businesses, while also powering 97 health facilities.

Three private sector companies came in to provide maintenance and operation, signing a public-private partnership agreement with the Government of Sierra Leone in December 2018.

“It was crucial to get away from the kind of install-and-forget approach taken in the past,” says Gonçalves. “Bringing in business has made the projects sustainable.”

While delighted to see these developments, as well as increased cross-sector activity, Gonçalves, like many experts, is concerned that investment in solar power still falls short of where it needs to be to advance universal health coverage and sustainable development goals.

“We are well short of what needs to be invested.”

Tamer Rabie

Dr Tamer Rabie, lead health specialist at the World Bank’s Health, Nutrition and Population Global Practice, concurs. “We are well short of what needs to be invested, given the scale of the challenge faced,” he says. To date, the Bank has invested US$ 300 million to support the electrification of almost 7500 health facilities in 27 countries. According to Rabie some US$ 5 billion is needed to bring reliable power to health facilities in 63 low- and middle-income countries.

Despite the fall in prices, the up-front cost of installing solar panels is still keeping some investors, including governments, on the side-lines. Vinci encourages a longer view. “At the beginning, the cost of a solar system and battery can be higher than a diesel generator, but if you calculate the cost of diesel over the 20-year life of a solar system, solar is much cheaper, even when you add the costs of the necessary operation and maintenance and battery replacement.”

Rabie agrees about the financial implications. “Governments need to plan for the long-term operation and maintenance costs, and find ways to integrate that into national budgets,” he says.

Like Gonçalves, Rabie sees public-private partnerships as essential to longer-term success. “In many initiatives, the private sector entity is at the heart of the model, running operations and in some cases establishing revolving funds to keep the system working, or selling surplus energy to communities or back to the government.”

Vinci too is supportive of private sector involvement, but flags limitations. “Where there is no viable market, the investment case comes down to the value of saving lives. Reliable electricity can make the difference between life and death, and should be ensured in every health-care facility.”

Whatever the approach taken, many countries have an abundance of sunlight and the potential to turn that sunlight into power.

“It has been estimated that Africa is home to 60% of the best solar resources worldwide,” says Vinci, “yet it only has 1% of installed solar photovoltaic capacity. There is clearly an opportunity to do more.”

Gonçalves agrees. “The sun is there,” she says. “If you really take solarization seriously and decide to do it, it's not going to be difficult.”

Having spearheaded efforts to get Shirati hospital a stand-alone solar system to use as a backup when grid electricity drops out, Bwire Chirangi could not agree more. “I thank God for the sun and the power it brings us every day,” he says. “It’s affordable and it’s clean. Now we have to use it.”

**Figure:**
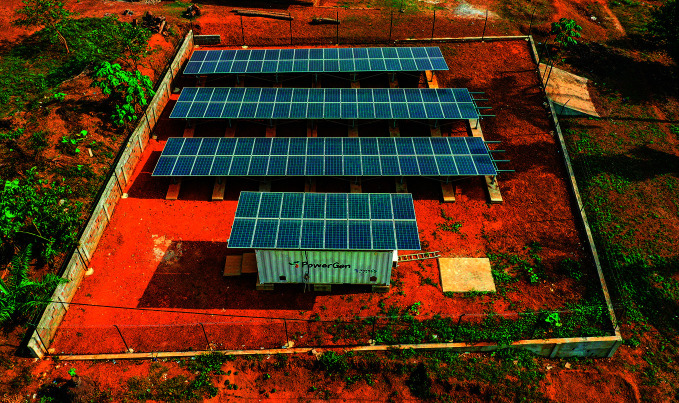
Photovoltaic panels installed as part of the rural renewable energy project in Sierra Leone

Courtesy of UNEP

**Figure:**
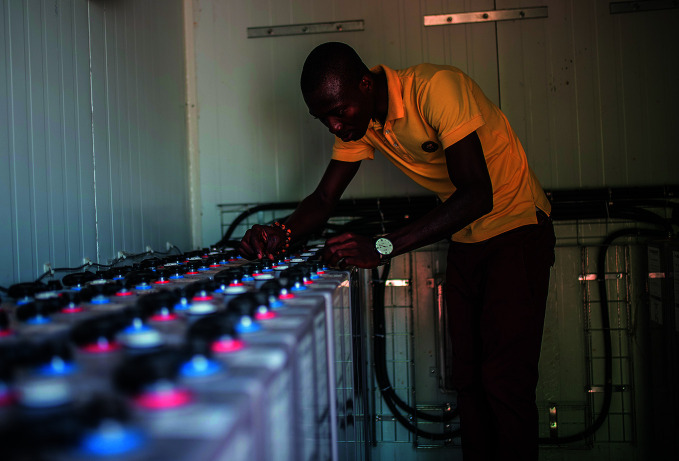
Checking batteries at a health facility in Sierra Leone.

Courtesy of UNEP

